# Simultaneous analysis of the expression of CD64 and HLA-DR in the peripheral blood and bronchoalveolar lavage fluid in sepsis

**DOI:** 10.1186/cc11944

**Published:** 2013-03-19

**Authors:** T Skirecki, M Mikaszewska-Sokolewicz, G Hoser, U Zielińska-Borkowska

**Affiliations:** 1 The Centre of Postgraduate Medical Education, Warsaw, Poland; 2 Medical University of Warsaw, Poland

## Introduction

The core pathophysiological changes in sepsis involve systemic activation of the immune system followed by the anti-inflammatory compensatory response. However, controversies exist regarding the status of the immune system in local tissue compartments during sepsis. The aim of this study was to compare selected markers of activation between the systemic circulation and local lung environment.

## Methods

Twenty patients with severe sepsis were included into this study. Peripheral blood (PB) samples and bronchoalveolar lavage fluid (BALF) samples were obtained on the day of diagnosis (D1). BALF was collected from 11 patients. Samples were stained with antibodies: CD15/CD64 and CD3/CD14/HLA-DR and isotypic control. Cells were analysed by flow cytometry. Expression of markers of activation was analysed as the geometric median of fluorescence (GMF). All values are expressed as median values. Comparisons between groups were performed using Mann-Whitney and Wilcoxon tests.

## Results

The mortality of sepsis reached 70%. Nonsurvivors had significantly (*P *= 0.001) elevated expression of CD64 on neutrophils. Expression of HLA-DR was higher in monocytes from BAL than PB GMF (1,032 vs. 342; *P *= 0.02) and this tendency was present in sepsis originating from both pneumonia and peritonitis. Percentage of HLA-DR-positive T cells was lower in PB than in BAL (2.9% vs. 6.5%; *P *= 0.07), but the GMF values for HLA-DR were higher in the circulating T cells (1,904 vs. 1,346; *P *= 0.004). The expression of CD64 on neutrophils was not significantly different in PB and BAL, but there was a trend towards its higher expression in BAL from patients with pneumonia while its expression was higher in PB of patients with peritonitis.

## Conclusion

In this study we noticed that during sepsis some significant differences in the status of activation of immune cells exist between peripheral blood and lung resident cells. The lung milieu seems to promote activation of monocytes while neutrophil activation is more dependent on the site of infection. However, these observations require further studies in a larger group of patients.

